# Presidential Symposium: National Institute of Aging P30 Results in AI and Technology for Healthy Aging and Persons With ADRD

**DOI:** 10.1093/geroni/igaf122.1521

**Published:** 2025-12-31

**Authors:** Pamela Cacchione, Elizabeth Vásquez, Pamela Cacchione

## Abstract

The National Institute on Aging funded three P30’s focused on Artificial Intelligence (AI) and Technology in ADRD and healthy Aging. University of Pennsylvania, Penn AITech, collaborating universities in Massachusetts, Mass AITC, and Johns Hopkins University, Hopkins AITC are in their last year of funding and have supported transdisciplinary 1 year pilot awards for 4 years for over 40 pilot awardees. This year completed these P30’s funding. The P30s are supported by the a2 collective a coordinating center dedicated to hosting the annual pilot award competition and enhancing communications across the three P30s. Each of the P30s have a different focus for their pilot funds and will share their successes and opportunities in running a P30. Each of the P30s funded pilot awards from industry, technology start-up companies, and academic institutions. Penn AI Tech focuses on technology and AI development to advance the science of caring for older adult or those with ADRD living in the community. Mass AITC spans 5 institutions and focuses on technologies and AI for successful aging and care of PLwD at home. Hopkins AITC focuses on the implementation of new AI and technology solutions to improve the health of older adults and those with ADRD with an emphasis on physical function. Each PI, George Demiris, PhD, (Penn AI Tech), Niteesh Choudhry, MD, PhD (Mass AITC), and Peter Abadir, MD, (Hopkins AITC) will describe their successful awardees’ accomplishments as well as challenges for the P30’s and the some of their pilot awardees over the first 3 years.

